# Assessing kidney donor quality: the saga continues

**DOI:** 10.1590/2175-8239-JBN-2025-E013en

**Published:** 2025-07-21

**Authors:** Tainá Veras de Sandes-Freitas

**Affiliations:** 1 Universidade Federal do Ceará, Complexo Hospitalar, EBSERH, Fortaleza, CE, Brazil.; 2 Universidade Federal do Ceará, Faculdade de Medicina, Fortaleza, CE, Brazil.

The Kidney Donor Risk Index (KDRI) is a composite risk score based on ten donor-specific variables developed to estimate the relative risk of graft failure following deceased donor kidney transplantation, using the ‘median donor’ of the previous calendar year as a reference. In the original publication, the median donor was defined as a 40-year-old, 1.70-meter tall, 80 kg, non-African American individual with brain death as the cause of death, no history of hypertension or diabetes, negative for hepatitis C virus, and without cerebrovascular cause of death. The Kidney Donor Profile Index (KDPI) is a percentile-based derivation of this model and positions a given donor relative to the overall donor pool^1^. This mathematical model was developed and validated in 2009 using data from the United States’ Scientific Registry of Transplant Recipients (SRTR); external validation in several countries outside the United States, including Brazil, is still pending.

Although KDPI provides a broader assessment of graft failure risk than the traditional classification of donors into standard or expanded criteria^2^, its predictive accuracy remains limited, with only moderate discriminative capacity for graft loss demonstrated in the original cohort and in external validation studies conducted in other countries^3^.

In this edition of the Brazilian Journal of Nephrology, Aquino de Morais AP et al. evaluated the association between KDPI and a composite outcome of graft loss, death, or estimated glomerular filtration rate (eGFR) by CKD-EPI below 30 mL/min/1.73 m^2^ at 1 year in a cohort of transplants from standard criteria donors. Although this was a single-center retrospective study, the sample of 1,943 patients was substantial^4^. As a result, KDPI was associated with the composite outcome. However, when analyzed separately, no association was found between KDPI and graft loss or patient death, but only with reduced renal function. Similar findings were previously reported by the same group in a cohort of 3,059 transplants that included both standard and expanded criteria donors^5^. These findings confirm the limitations of using this score in our population to predict graft loss, the outcome for which it was originally developed.

Among the challenges in externally ­validating this predictive score are the ­variables it includes: donor age, height, weight, ethnicity, history of hypertension or diabetes, cause of death, final serum creatinine, hepatitis C serostatus, and type of death (brain death or circulatory death). In Brazil, race/ethnicity assessment is particularly complex, as a large proportion of the population is multiracial. Additionally, most transplant ­centers do not use HCV-positive donors, and donation after circulatory death is not allowed in our country. Furthermore, information on prior comorbidities such as hypertension and diabetes are often inaccurate, particularly with regard to disease duration. Finally, in our setting, final creatinine levels often reflect acute kidney injury, which is not associated with long-term graft loss^6^. This helps explain why donor age alone has shown a discriminatory ability comparable to that of KDPI in some studies, making the routine use of KDPI an exception in most Brazilian centers, particularly in the current context where an online KDPI calculator is not available^7^.

It would have been highly valuable to present the results of the sub-analysis of the two cohorts – standard and expanded criteria donors – to allow a comparison of KDPI’s predictive performance across these subgroups. The predictive capacity may be greater in the standard criteria donor group due to the greater age variability within this population^7^.

In summary, the search continues for a non-binary predictor of graft loss that can reliably assess kidney graft quality and provide a greater discriminatory power than donor age alone. To date, there is no evidence that decisions to accept or reject donor kidneys should be made solely on the basis of a single predictor such as KDPI. Instead, such decisions should be based on a combination of variables, including donor demographic and clinical data, graft histology when available, and recipient-specific clinical and demographic characteristics.

## Data Availability

No new data was generated or analyzed in this study.
