# Supplementary material for: Assessing kidney donor quality: the saga continues
Source: J Bras Nefrol. 2025 Jul 21;47(3):e2025E013. doi: 10.1590/2175-8239-JBN-2025-E013en (PMC12296198; doi:10.1590/2175-8239-JBN-2025-E013en)
Supplement: Translation

# Translation: Avaliação da qualidade do doador renal: a saga continua

**DOI:** 10.1590/2175-8239-JBN-2025-E013pt

**Authors:** Tainá Veras de Sandes-Freitas

**Affiliations:** 1 Universidade Federal do Ceará, Complexo Hospitalar, EBSERH, Fortaleza, CE, Brasil.; 2 Universidade Federal do Ceará, Faculdade de Medicina, Fortaleza, CE, Brasil.

O *Kidney Donor Risk Index* (KDRI) é um escore de risco composto por dez variáveis do doador, desenvolvido com o objetivo de estimar o risco relativo de perda do enxerto após um transplante com doador falecido, usando como referência o “doador mediano” do ano anterior. Na publicação original, esse doador era representado por um indivíduo com morte encefálica, não afro-americano, 40 anos, 1,70 m de altura, 80 kg, sem antecedentes de hipertensão ou diabetes, negativo para o vírus da hepatite C e que não teve morte por causa cerebrovascular. O *Kidney Donor Profile Index* (KDPI) é uma derivação em percentil desse modelo, posicionando o doador em questão em relação ao grupo total de doadores^1^. Esse modelo matemático foi elaborado e validado em 2009, utilizando dados do registro norte-americano *Scientific Registry of Transplant Recipients* (SRTR), e até o momento ainda carece de validação externa em vários países fora dos Estados Unidos, incluindo o Brasil.

Apesar de fornecer uma avaliação de risco de perda do enxerto menos binária do que a tradicional classificação dos doadores em critério padrão e critério expandido^2^, a acurácia na predição de desfechos é limitada, com apenas moderada capacidade discriminante para predição de perda do enxerto, demonstrada tanto na coorte original quanto em estudos de validação externa realizados em outros países^3^.

Nesta edição do *Brazilian Journal of Nephrology*, Aquino de Morais AP e colaboradores avaliaram a associação entre o KDPI e o desfecho composto de perda do enxerto, óbito ou taxa de filtração glomerular estimada (TFGe) por CKD-EPI inferior a 30 mL/min/1,73 m^2^ ao final de 1 ano, em uma coorte de transplantes com doadores de critério padrão. Trata-se de um estudo unicêntrico e retrospectivo; porém, com uma amostra bastante significativa, composta por 1943 pacientes^4^. Como resultado, o KDPI foi associado ao desfecho composto. No entanto, a análise dos desfechos separadamente demonstrou não haver associação entre KDPI e perda do enxerto ou óbito, mas apenas com função renal inferior, o que foi previamente demostrado pelo mesmo grupo em uma coorte de 3059 transplantes, incluindo doadores de critério padrão e expandido^5^. Esses resultados confirmam a dificuldade de utilização desse escore em nossa população como um preditor de perda do enxerto – desfecho para o qual ele foi originalmente desenvolvido.

Dentre as dificuldades de validação externa do preditor estão as próprias variáveis que o compõem: idade do doador, altura, peso, etnia, história de hipertensão arterial ou diabetes, causa do óbito, creatinina sérica final, sorologia para hepatite C e tipo de morte (encefálica ou cardíaca). No Brasil, a avaliação da etnia/raça é bastante complexa, uma vez que boa parte da população é multirracial. Além disso, a maioria dos centros não realiza transplantes com doadores HCV positivo, e a doação com coração parado não é prevista no país. Adicionalmente, a informação sobre comorbidades prévias, como hipertensão e diabetes, nem sempre é acurada, principalmente quanto à sua duração. Por fim, a creatinina final, em nossa realidade, geralmente reflete situações de injúria renal aguda, a qual não tem associação com perda do enxerto no longo prazo^6^. Isso justifica por que a idade do doador tem demonstrado poder discriminante semelhante ao do KDPI em alguns estudos, tornando seu uso rotineiro uma exceção na maioria dos centros brasileiros, sobretudo atualmente, em que não dispomos de uma calculadora de KDPI online^7^.

Teria sido de grande valia a apresentação dos resultados da subanálise das duas coortes – doadores de critério padrão e de critério expandido – a fim de que pudéssemos comparar a capacidade do KDPI em predizer desfechos nesses subgrupos de forma separada. É possível que a capacidade preditiva seja melhor na amostra de doadores de critério padrão em virtude da maior variabilidade de idade observada nessa população^7^.

Em suma, a busca por um preditor de perda do enxerto não binário, capaz de avaliar a “qualidade” de um rim para transplante e que agregue capacidade discriminante significativamente superior à da idade do doador isoladamente, ainda persiste. Até o momento, as evidências disponíveis não suportam que a decisão por utilizar ou descartar rins para transplante seja baseada exclusivamente em um preditor como o KDPI. Essa decisão deve considerar um conjunto de variáveis que envolvam os dados demográficos e clínicos do doador e a histologia do enxerto, se disponível, bem como os dados demográficos e clínicos do receptor.

## Disponibilidade de Dados

Nenhum dado novo foi gerado ou analisado neste estudo.
